# Post-obstructive Acute Kidney Injury Due to a Retroperitoneal Mass

**DOI:** 10.7759/cureus.82007

**Published:** 2025-04-10

**Authors:** Zoraize Moeez Athar, Mahnoor Arshad, Gabrielle Davis, Jacob Epperson, Kalpana A Uday

**Affiliations:** 1 Internal Medicine, BronxCare Health System, New York City, USA; 2 Internal Medicine, American University of the Caribbean, Cupecoy, SXM; 3 Medicine, American University of the Caribbean, Cupecoy, SXM; 4 Nephrology, BronxCare Health System, New York City, USA; 5 Medicine, Icahn School of Medicine at Mount Sinai, New York City, USA

**Keywords:** acute kidney injury (aki), benign, neurofibromatosis type 1, nf1, pelvic mass, post obstructive aki, postrenal aki, retroperitoneal mass, young patient

## Abstract

This case report discusses post-obstructive acute kidney injury (AKI) in a 26-year-old woman with neurofibromatosis type 1 (NF1) and uterine fibroids. The patient's AKI was diagnosed through elevated creatinine and blood urea nitrogen (BUN) levels and confirmed by transabdominal ultrasound and computed tomography (CT) scan, which revealed severe bilateral ureterohydronephrosis. The patient underwent fluoroscopic and sonographic bilateral nephrostomy tube placement, which significantly improved renal function. An exploratory laparotomy and superficial mass biopsy indicated a retroperitoneal tumor consisting of fibroadipose and muscular tissue with focal suppurative inflammation. The patient's renal function normalized by discharge. This case underscores the importance of early recognition, radiological evaluation, and timely intervention in managing AKI secondary to obstructive uropathy and calls for further research on optimal management strategies for AKI secondary to primary retroperitoneal masses.

## Introduction

Acute kidney injury (AKI), previously known as acute renal failure, is defined by a sudden and rapid decline in kidney function. This condition occurs over a short period, typically within a few hours to a few days. As defined by the Kidney Disease Improving Global Outcomes (KDIGO), it is diagnosed by a rise in serum creatinine either 0.3 mg/dL (milligrams per deciliter) or more in a period of 48 hours or an increase by 1.5 times its baseline value. It can also be diagnosed by a decreased urine output of less than 0.5 ml/kg/hr (milliliters per kilogram per hour) in a period of six to 12 hours [1, 2].

Acute kidney injury can be differentiated into prerenal, intrarenal, and postrenal etiologies, each having overlapped and interrelated pathophysiologies. The prerenal form of AKI can be due to anything that reduces blood flow to the kidney. This may be due to systemic hypoperfusion from hemorrhage or decreased blood flow from renal artery stenosis [3]. Intrinsic renal causes include conditions that affect the glomerulus or renal tubules, such as acute tubular necrosis or interstitial nephritis, although prolonged prerenal injury can cause intrarenal injury if the precipitating factors are prolonged enough to cause cellular damage [3]. The postrenal form of AKI includes obstructive causes, leading to urinary backflow in the filtration system. Disturbances of the urinary outflow tract can cause pathologic signs within two hours of obstruction. This results in decreased renal perfusion, inflammation, tubular atrophy, and interstitial fibrosis [2]. If the blockage remains for a long enough time, it can progress to end-stage renal disease, but in most situations, once the obstruction is released, renal function slowly progresses to normal [3].

Current diagnostic approaches to AKI are based on acute changes in glomerular filtration rate (GFR), indicated by an acute rise in serum creatinine. According to KDIGO staging criteria, it is important to quantify, as it directly correlates with higher mortality and longer hospital stays [1,4]. This staging system is based on a modified version of the classification by the Risk, Injury, Failure, Loss, and End Stage Kidney Disease (RIFLE) criteria, which separates the severity grading (risk, injury, and loss) and outcome grading (loss and end stage kidney disease) [5].

While the causes of AKI are broad and often identifiable through clinical context and laboratory tests, some cases present with atypical or obscure underlying etiologies that complicate diagnosis and management. Structural abnormalities, including obstructive lesions from rare retroperitoneal tumors, may lead to postrenal AKI and often evade early detection due to their deep anatomical location and nonspecific symptomatology. These rare causes emphasize the importance of a thorough diagnostic approach, especially when initial evaluations fail to reveal a clear etiology.

Primary retroperitoneal masses are exceptionally rare, accounting for 0.1% to 0.2% of all neoplasms [6]. These neoplasms can be difficult to diagnose and are often mistaken for uterine or ovarian pathology in females and are often only diagnosed after incidental findings or after presentation of urinary or gastrointestinal symptoms [7]. Displacement of intraperitoneal and retroperitoneal organs helps clarify the location of a primary retroperitoneal tumor when evaluated through radiographic and radiofrequency imaging. These tumors pose significant diagnostic and therapeutic challenges due to their location. Such masses are often inaccessible during surgical approaches as well and have a delayed and indolent presentation. We report a case of post-obstructive AKI secondary to a rare primary retroperitoneal mass. 

## Case presentation

A 26-year-old woman with a known past medical history of neurofibromatosis type 1 (NF1) and uterine fibroids was admitted to the hospital with complaints of abdominal pain worsening over a period of four weeks with associated symptoms of nausea, non-bloody, non-bilious vomiting, constipation, dysuria, urinary urgency, and urinary frequency, and approximately 18 kg (kilograms) weight loss. There were no complaints of any fever or hematuria.

Vital signs upon presentation were within normal limits except her blood pressure was elevated to 170/100 mmHg (millimeters of mercury). Her physical exam revealed the presence of abdominal distension.

Lab values were significant for elevated creatinine and blood urea nitrogen (BUN) levels and decreased GFR, indicating AKI. In simple terms, GFR and creatinine are inversely related; a lower than expected value of GFR signifies declining kidney function. Blood urea nitrogen, while not part of the diagnostic criteria for AKI, does provide important information when viewed together with other laboratory values in the etiology of AKI and can help differentiate between prerenal, intrinsic, and post-obstructive AKI. The patient’s renal function tests over the course of her hospitalization are displayed in Table 1, comparing them to baseline values for the same patient almost one year prior to the current admission.

**Table 1:** Renal function tests mg/dL: milligram per deciliter; mL/min/1.73m^^^2: milliliters per minute per 1.73 square meters

Laboratory test	Baseline	On admission	Two days after admission	After nephrostomy tube placement	On discharge day	Normal value
Blood urea nitrogen (mg/dL)	9	41	44	31	17	6-20
Creatinine (mg/dL)	0.5	2.9	4.1	2.5	0.7	0.5-1.5
Glomerular filtration rate (mL/min/1.73m^2)	171.75	20.58	14.02	25.04	107.64	>90

**Figure 1:**
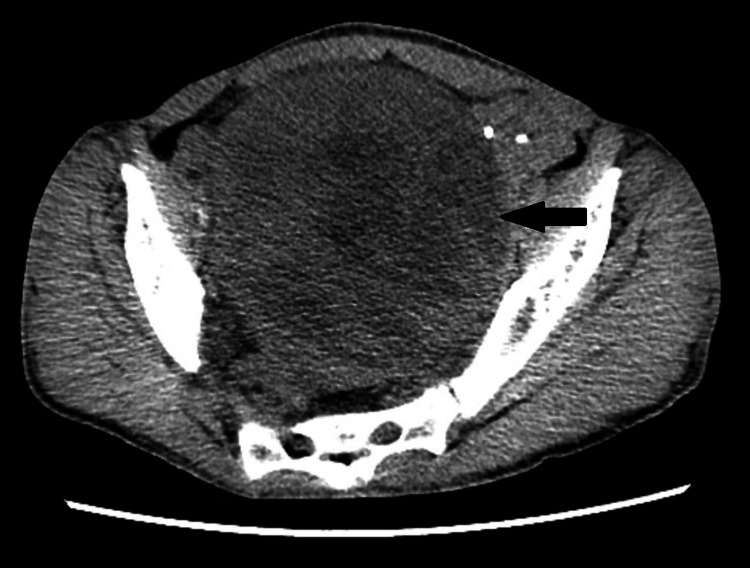
A CT scan of the pelvis (axial view) The black arrow points to the mass.

**Figure 2:**
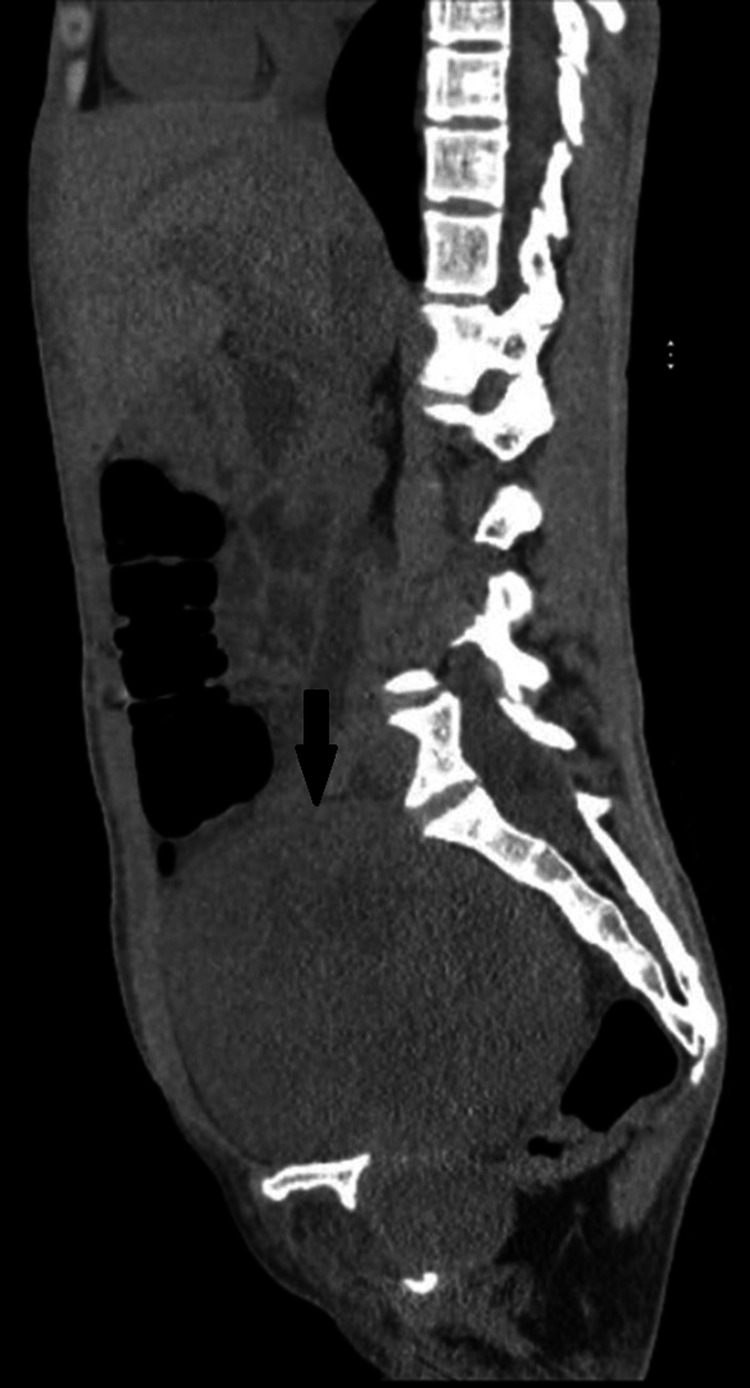
A CT scan of the abdomen and pelvis (sagittal view) The black arrow points to the pelvic mass.

Due to these findings, the patient underwent a transabdominal ultrasound focused on the kidneys, which is a common and safe diagnostic test to perform after there is derangement of renal function tests noted, the findings of which revealed the patient to have severe bilateral ureterohydronephrosis. The presence of hydronephrosis could signify an obstruction causing reflux of urine, which would require further imaging to evaluate the etiology. This prompted a computerized tomography (CT) scan of the abdomen and pelvis without contrast material (Figures 1, 2). This test confirmed the presence of ureterohydronephrosis in both kidneys. Additionally, it revealed a mass in the mid-pelvis extending down along the right inferior pelvic sidewall. The CT scan reported that the presence of hydronephrosis was secondary to this mass. 

**Figure 3:**
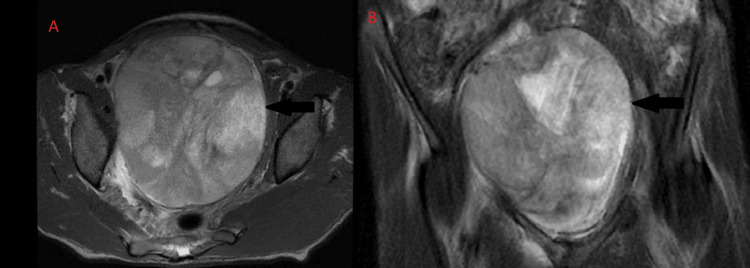
Transverse (panel A) and coronal view (panel B) of the MRI The black arrow points to the mass.

**Figure 4:**
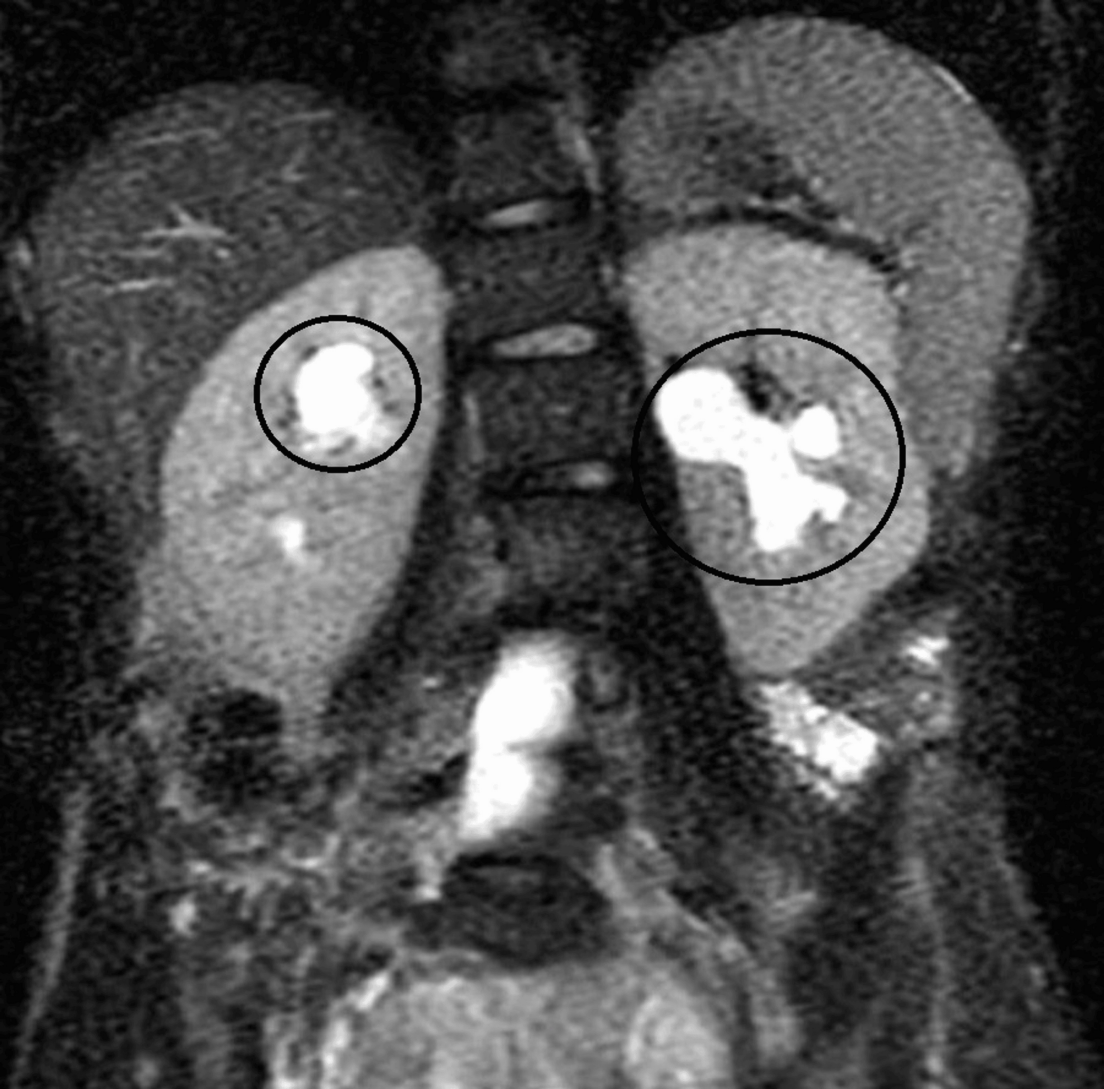
Coronal view of the MRI of the kidneys showing presence of hydronephrosis. Dilated renal calyces are circled.

An MRI of the abdomen and pelvis without contrast was performed after the CT to have a clearer picture of the mass, since an MRI is a better imaging modality than a CT scan when evaluating soft tissue structures of the body. This revealed bilateral hydronephrosis and a large heterogeneous mass occupying much of the pelvis, measuring 12.3 cm x 11.0 cm x 12.5 cm (Figures 3, 4). The endometrial complex was not well evaluated secondary to mass effect. The right and left ovaries are not clearly identified, since they are likely displaced. No lymphadenopathy, no free fluid in the peritoneum, and no peritoneal abnormalities were seen. Visualized pelvic bowel loops were normal in caliber without obstruction or inflammatory changes. There was no bladder wall thickening. 

The patient underwent fluoroscopic and sonographic bilateral nephrostomy tube placement. Her renal function tests improved dramatically just one day after nephrostomy tube placement (Table 1).

**Figure 5:**
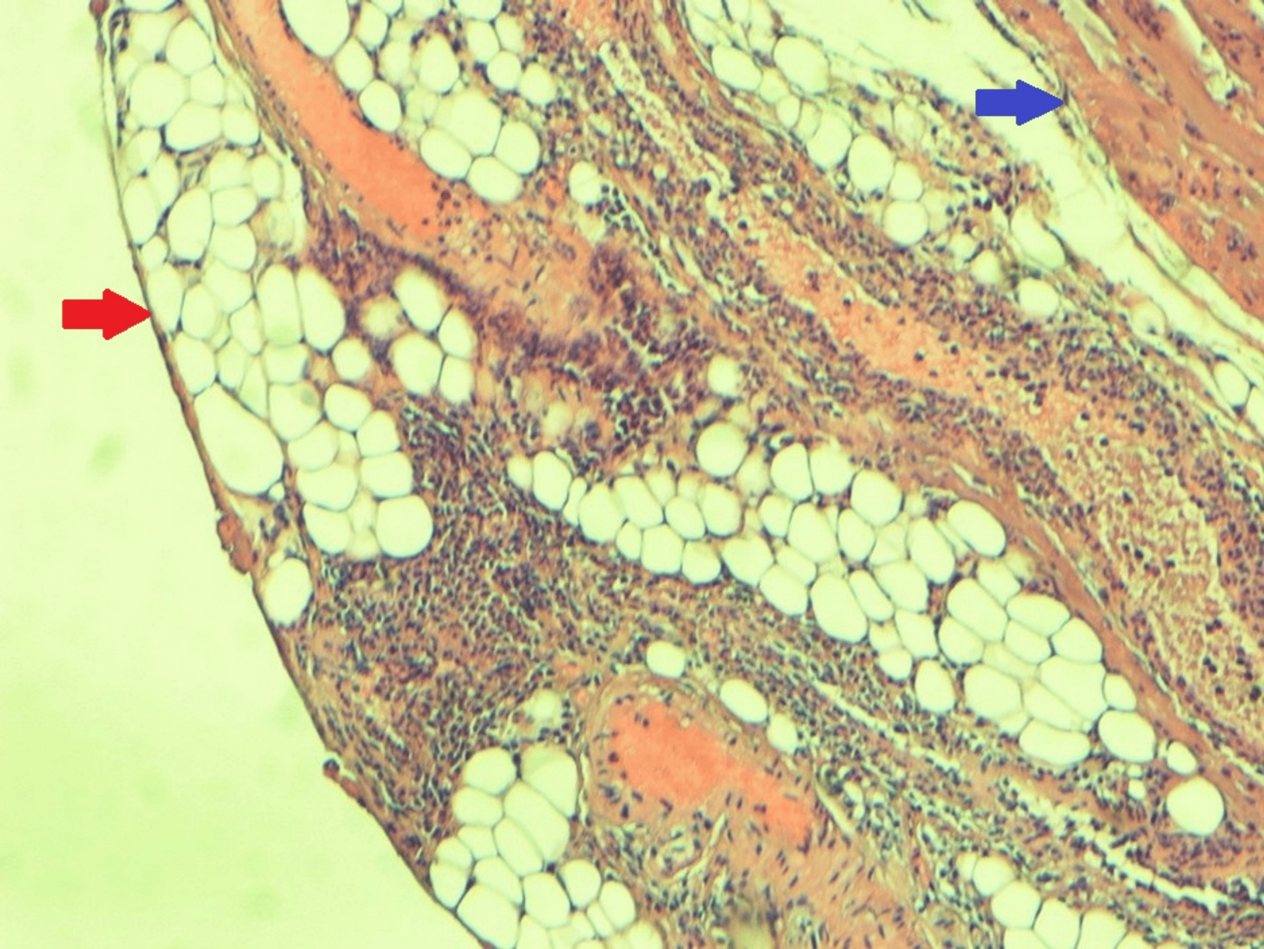
Histology of the mass The red arrow points to adipose tissue. The blue arrow points to the muscle tissue.

One week after nephrostomy tube placement, she underwent exploratory laparotomy with superficial mass biopsy. During laparotomy, the mass was found to be firm and fixed, arising from retroperitoneal structures, distinct from the uterus. A 1 cm x 1.5 cm margin of tissue was excised for biopsy. No further deep biopsy was performed as the tumor appeared very vascular to the surgical team. Biopsy results indicated a retroperitoneal tumor consisting of fibroadipose and muscular tissue showing focal suppurative inflammation with no evidence of cellular atypia or neoplasia (Figure 5). 

The patient was deemed stable and discharged 11 days after admission. On the day of discharge, AKI resolved completely (Table 1). 

## Discussion

In this case, a rare instance of post-obstructive AKI secondary to a primary retroperitoneal mass in a young woman with a history of NF1 and uterine fibroids was detailed. Upon presentation, the patient reported a four-week history of abdominal pain, nausea, vomiting, constipation, dysuria, urinary urgency, and significant weight loss. These clinical manifestations, coupled with physical examination findings of abdominal distention secondary to a pelvic mass, suggested an underlying pathological process. Furthermore, the patient's elevated blood pressure on presentation may have been indicative of concomitant acute renal dysfunction. As per the KDIGO staging criteria, the patient’s serum creatinine and estimated glomerular filtration rate (eGFR) indicated that she developed stage 3 kidney injury [4]. 

Radiological imaging, including transabdominal ultrasound and CT scan, played a crucial role in identifying the underlying cause of the patient's AKI. Severe bilateral ureterohydronephrosis secondary to a large pelvic mass was confirmed, and prompt intervention with bilateral nephrostomy tube placement led to a rapid improvement in renal function until reaching her normal baseline by discharge on day 11. Surgical exploration via exploratory laparotomy provided additional information on the nature of the retroperitoneal mass. The firm, fixed mass arising from retroperitoneal structures, distinct from the uterus, suggested a primary retroperitoneal origin. Histopathological analysis of superficial mass biopsy samples revealed a retroperitoneal tumor consisting of fibroadipose and muscular tissue with focal suppurative inflammation, ruling out neoplastic etiologies at this stage. 

Although this case demonstrates a transient AKI with complete resolution, a further analysis of renal function in NF1 patients, through a study comparing renal function rebound in NF1 patients to BMI-matched control patients, revealed that the BUN-to-creatinine ratio, a common marker for AKI, was significantly higher in NF1 patients compared to controls [8]. However, since the study excluded patients with acute diseases, including those with acute renal damage, the results provide insight into potential underlying factors for the elevated ratio in NF1 patients. Possible explanations include a higher protein intake leading to increased BUN values, a protein-hypercatabolic state causing elevated BUN, reduced muscle mass resulting in lower creatinine levels, and dehydration [8].

While previous studies have shown that NF1 patients often have reduced muscle size, strength, and inadequate nutrient intake, it is unlikely that protein overconsumption is the cause, making the other factors more plausible. Additionally, renal function was notably worse in male NF1 patients with a history of hypertension compared to those without, suggesting that hypertension, potentially due to renal artery stenosis or other pathology, worsens renal function in this population [8]. Interestingly, the overall prevalence of hypertension did not significantly differ between NF1 patients and controls, implying that hypertension alone does not explain the observed differences in renal function. In this study, renal function was assessed using creatinine-based eGFR, a common tool that is influenced by factors such as age, sex, and muscle mass. While creatinine clearance offers a more precise measure of renal function, eGFR remains a practical screening tool in such populations.

Renal impairment in NF1 is often associated with vascular issues like renal artery stenosis, which is a leading cause of death in NF1 patients after malignancies [9,10]. Hydroureteronephrosis affects 60% to 70% of NF1 patients and can lead to kidney atrophy and chronic kidney disease (CKD) in up to 40% of cases, with 8% eventually progressing to end-stage renal disease [9]. The combination of these findings illustrates the multifaceted nature of renal impairment in NF1 and the importance of vigilant monitoring and early intervention to prevent further renal complications.

The prognosis for AKI secondary to NF1 is difficult to measure but is often considered to be good, often dependent on the extent of periureteral obstruction/fibrosis [11]. With limited data on predictors of response to therapy or relapse, without mechanical intervention, i.e., stent placement, the recurrence rate has been reported as high as 70%, and with intervention as high as 10% [12].

Our patient’s favorable clinical outcome, including the resolution of AKI and normalization of renal function at discharge, underscores the importance of a multidisciplinary approach, combining radiological, surgical, and medical interventions, in managing complex cases of AKI secondary to obstructive uropathy. This case also highlights the critical role of timely diagnosis and management in preventing irreversible renal damage.

## Conclusions

This case highlights the diagnostic and therapeutic challenges associated with primary retroperitoneal masses presenting with obstructive AKI. The early diagnosis and treatment of severe ureterohydronephrosis, along with timely surgical biopsy and medical interventions, were pivotal in restoring the patient’s renal function and preventing long-term damage. This case highlights that, while NF1 patients may face unique renal challenges, a multidisciplinary approach can significantly improve outcomes, underscoring the need for heightened awareness and proactive management in such complex cases. Further studies are warranted to elucidate the optimal management strategies for AKI secondary to primary retroperitoneal masses, particularly in the context of underlying predisposing conditions such as NF1.
